# Supplementary figures and images for: Whole genome sequencing identifies novel structural variant in a large Indian family affected with X-linked agammaglobulinemia
Source: PLoS One. 2021 Jul 12;16(7):e0254407. doi: 10.1371/journal.pone.0254407 (PMC8274882; doi:10.1371/journal.pone.0254407)

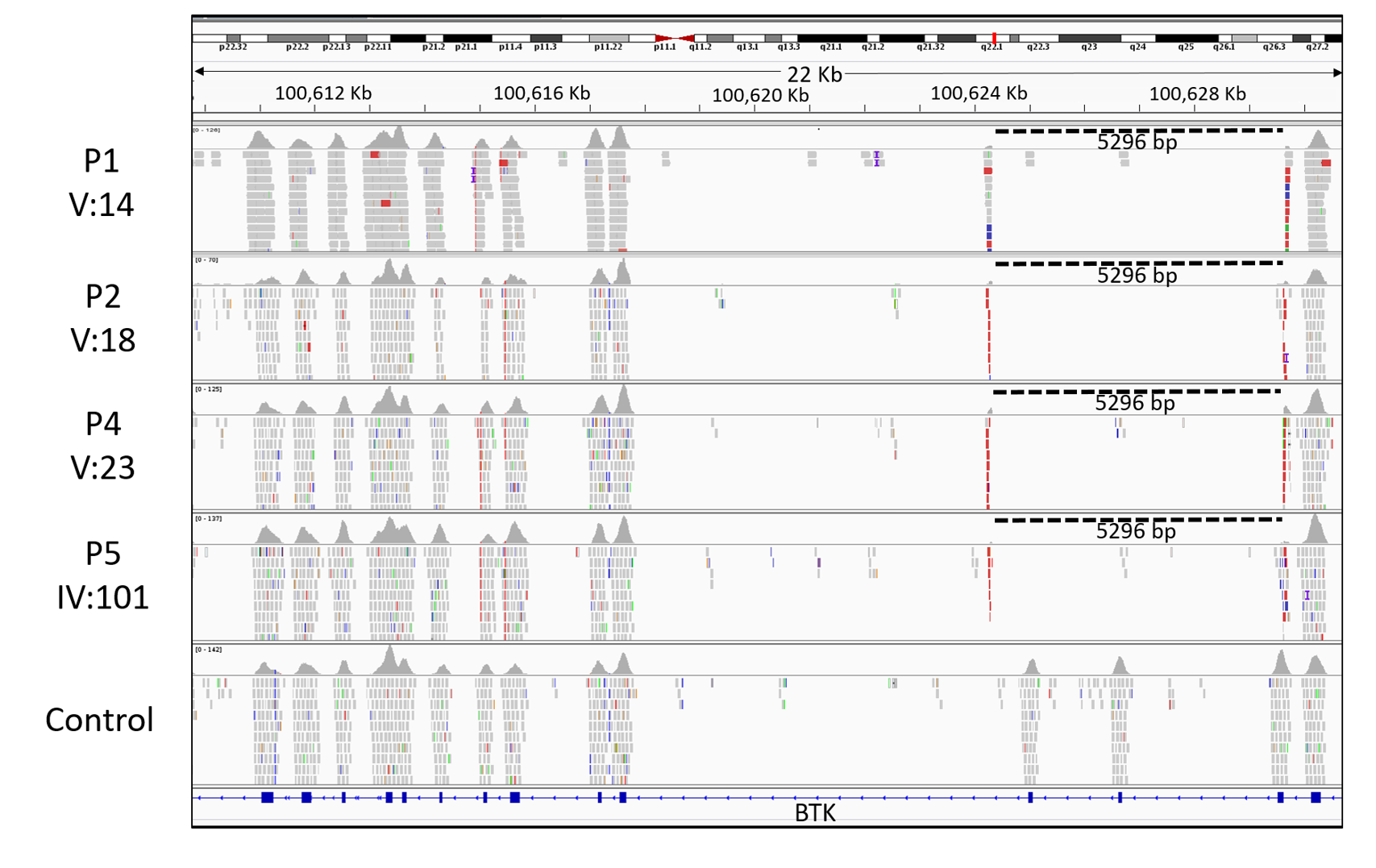

Supplement: S1 Fig — (TIF) [file pone.0254407.s001.tif]

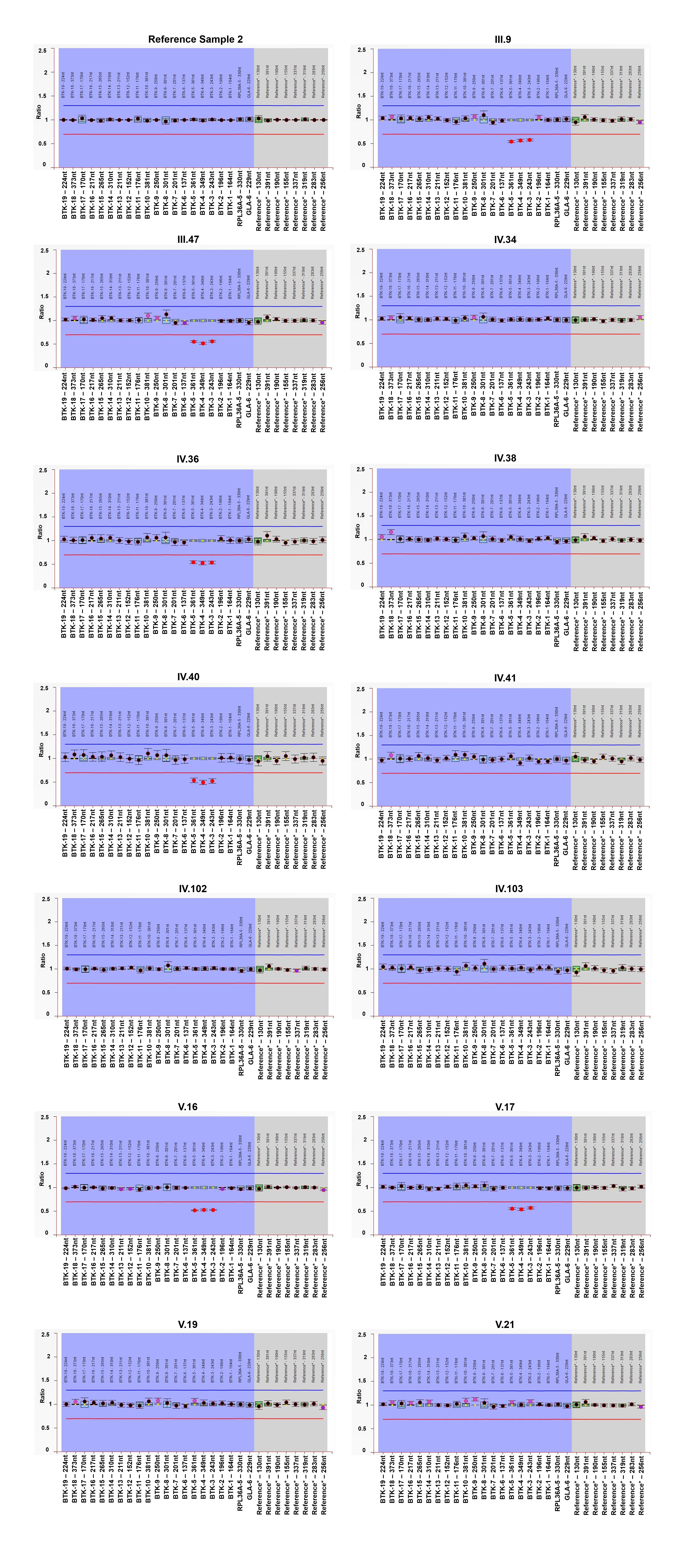

Supplement: S2 Fig — Representation of ratio charts generated using the Coffalyser.net software. Longitudinal axis represents the final ratios after inter and intra normalisations of the probe ratios. Horizontal axis represents the probe names along with the length (The axis titles have been manually enlarged for clarity). The blue and red horizontal lines depict the arbitrary borders of ratio 1.3 and 0.7 respectively. The black and red dots denote the final ratio obtained for each of the probes and the vertical bars represent the 95% confidence range for each probe. The test probes are of BTK gene and the rest are the reference probes. The Roman and numeric numbers on top of each ratio chart represent the individual marked as per the pedigree in Fig 1. (TIF) [file pone.0254407.s002.tif]

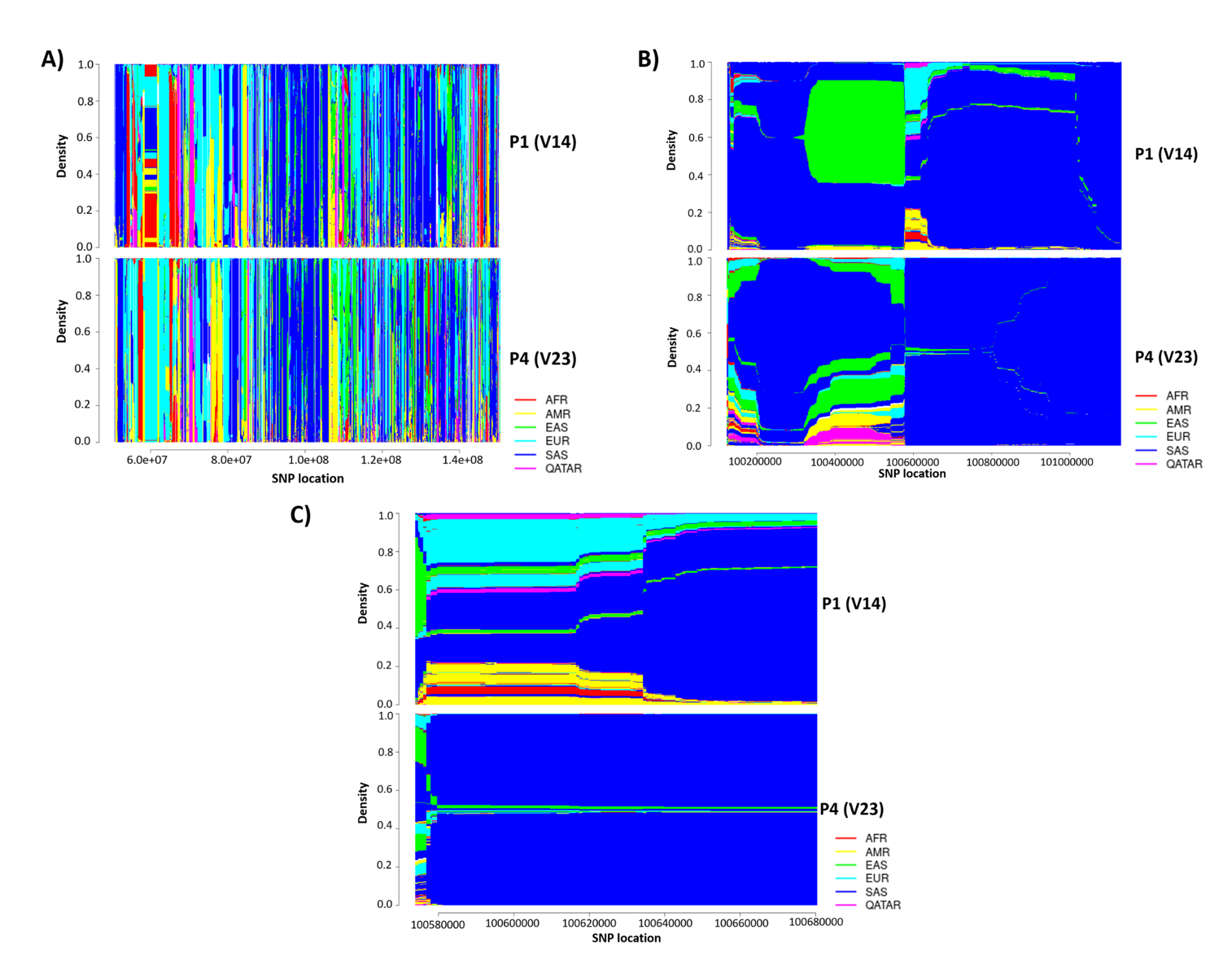

Supplement: S3 Fig — A) 50MB upstream and 5KB downstream, B) 500KB upstream and 500KB downstream, and C) 50KB upstream and 50KB downstream to loci chrX:100,624,323–100,629,619 (hg19/GRCh37) of two affected first cousins (V14 and V23) with 2504 individuals of five major populations (AFR-African, AMR- American, EAS- East Asian, EUR-European, and SAS-South Asian) of 1000 Genome Project and 44 individuals of Qatar ancestry. (TIF) [file pone.0254407.s003.tif]
