# Supplementary material for: Whole genome sequencing identifies novel structural variant in a large Indian family affected with X-linked agammaglobulinemia
Source: PLoS One. 2021 Jul 12;16(7):e0254407. doi: 10.1371/journal.pone.0254407 (PMC8274882; doi:10.1371/journal.pone.0254407)
Supplement: S1 Table — (PDF) [file pone.0254407.s004.pdf]

Data	P1	P2	P4	P5
**Total number of reads (x2)**	39111154	41867264	37556352	41367110
**Trimmed reads (x2)**	22325405	31637375	18905832	36276744
**Reads mapped (x2)**	22320244	31616422	18851742	36276174
**Percentage mapped**	99.08%	99.30%	99.71%	100%
**Coverage**	74.4	105.4	62.8	120.9
**Total variants**	359,707	647,936	328,688	534,222
**Protein altering variants**	12,219	15,304	11,809	12,966
**Rare variants (MAF < 5%)**	1,552	4,185	2,193	1,602
**PID genetic variants (454 gene)**	9	9	17	16
**Phenotype associated variant**	0	0	0	0
**Machine Used**	HiSeq2500	HiSeq2500	HiSeq2500	NovaSeq6000
**Library Used**	TruSeq DNA Exome	TruSeq DNA Exome	TruSeq DNA Exome	TruSeq DNA Exome

S1 **Table:** Whole exome sequencing data summary for each patient.
