# Supplementary material for: Whole genome sequencing identifies novel structural variant in a large Indian family affected with X-linked agammaglobulinemia
Source: PLoS One. 2021 Jul 12;16(7):e0254407. doi: 10.1371/journal.pone.0254407 (PMC8274882; doi:10.1371/journal.pone.0254407)
Supplement: S3 Table — The roman and numeric numbers on top of each ratio chart represent the individual marked as per the pedigree in Fig 1. (PDF) [file pone.0254407.s006.pdf]

		Reference sample 1	Reference sample 2	III.9	III.47	IV.34	IV.36	IV.38	IV.40	IV.41	IV.101	IV.102	IV.103	V.14	V.16	V.17	V.18	V.19	V.21	V.23
	BTK-19 - 224nt	1	1	1.04	1.02	1.03	1.03	1.06	1.03	0.98	1.06	1.01	1.05	1.08	0.99	1.02	1.12	1	1.02	1.04
	BTK-18 - 373nt	1	1	1.07	1.05	1.05	1.01	1.16	1.08	1.08	1.05	0.99	1.03	1.04	1.02	1	0.98	1.06	1.06	0.97
	BTK-17 - 170nt	0.97	1.03	1.03	1.02	1.06	1.01	1.02	1.07	1.01	1.04	1	1.01	1	1	1.03	1.04	1.06	1.03	0.99
	BTK-16 - 217nt	1	1	1.04	1.01	1.03	1.06	1.02	1.03	1.01	1.06	1.01	1.04	1.11	1.01	1	1.11	1.04	1.05	1.06
	BTK-15 - 265nt	1.02	0.98	1.03	1.05	1.01	1.04	1	1.03	1.07	1.02	0.98	0.97	0.95	0.98	1.02	1.01	1.02	1.05	0.98
	BTK-14 - 310nt	0.99	1.01	1.06	1.05	1	1.06	0.99	1.07	1.02	1.09	1.01	0.99	1.04	0.99	0.97	1.03	1.03	1.07	0.96
	BTK-13 - 211nt	1	1	1.03	1	1.02	1	1.02	1.03	0.98	1	1.02	1.01	1	0.97	0.98	1.06	0.99	1	1
	BTK-12 - 152nt	1	1	1	1.01	1.02	0.98	1.01	0.99	1.02	1.02	1.03	1.01	1	0.97	1.02	1.07	0.98	0.99	1.05
	BTK-11 - 176nt	0.97	1.03	0.96	1.01	0.99	0.98	0.99	0.97	1.09	0.94	0.97	0.94	1.01	0.96	1.03	0.94	0.97	1.03	0.93
	BTK-10 - 381nt	1.02	0.98	1.03	1.1	1.04	1.07	1.07	1.11	1.09	1.05	1.01	1.07	1.07	0.99	1.05	1.02	1.06	1.09	1
	BTK-9 - 250nt	1	1	1.07	1.06	1.06	1.06	1.03	1.07	1.05	1.11	1	1.02	1.08	1	1.03	1.04	1.08	1.12	0.97
	BTK-8 - 301nt	1.03	0.97	1.1	1.13	1.06	1.07	1.07	1.07	0.99	1.12	1.08	1.11	1.08	1.01	1.04	1.11	1.03	1.05	1.1
	BTK-7 - 201nt	1.01	0.99	0.95	0.95	1.01	0.97	1.01	0.97	0.98	0.96	1.01	1	1.01	1	0.97	1.01	0.97	0.97	0.98
	BTK-6 - 137nt	1	1	0.99	0.95	0.99	0.96	0.99	0.98	1	0.96	1.02	1.02	1.01	1	1	1.05	0.96	0.98	1.01
	BTK-5 - 361nt	0.99	1.01	0.54	0.55	1.01	0.54	1.05	0.54	1.06	0	1	1.01	0	0.52	0.56	0	1.08	1.06	0
	BTK-4 - 349nt	1.01	0.99	0.57	0.52	1.02	0.53	1.01	0.49	0.92	0	1.01	1.03	0	0.53	0.54	0	1.03	1.01	0
	BTK-3 - 243nt	0.99	1.01	0.58	0.56	1	0.54	0.97	0.52	1.02	0	1.02	0.98	0	0.53	0.57	0	1.02	1.08	0
	BTK-2 - 196nt	1	1	1.05	0.99	1.01	1.04	1.02	1.01	0.95	1.05	1	1.03	1.1	0.97	0.99	1.11	1.02	1.02	1.04
	BTK-1 - 164nt	1	1	1	0.99	1.03	1.01	1.03	1.02	0.95	1.03	1	1.03	1.05	0.99	0.98	1.08	1.01	1	1.04
	RPL36A-5 - 330nt	0.98	1.02	1.01	1	1	1	0.95	0.99	1	0.92	0.96	0.95	1.07	0.97	0.98	0.93	1	1.02	0.95
**Test probes**	GLA-6 - 229nt	0.98	1.02	1.02	0.96	1	1.03	0.97	0.97	0.97	0.96	1	0.97	1.01	0.98	0.99	1.01	0.99	0.98	0.99
	Reference - 130nt	0.97	1.03	0.95	0.98	1	0.98	0.99	0.94	1.05	0.95	0.98	0.93	1.05	0.97	1	0.95	0.96	1.01	0.98
	Reference - 391nt	1.01	0.99	1.06	1.07	1.01	1.11	1.06	1.04	0.98	1.09	1.06	1.06	1.03	1.02	0.99	1.05	1.02	1.07	1.04
	Reference - 190nt	1	1	1.01	1.03	1.02	1.03	1.03	0.95	0.95	1.03	1	1.02	1.05	1	1.01	1.02	0.99	1	1.02
	Reference - 155nt	1	1	0.99	1	0.97	0.95	0.98	1.05	1.05	1	0.98	0.97	0.98	1.01	0.99	1	1.04	1	0.97
	Reference - 337nt	1	1	0.98	1	1	0.98	1.01	0.99	1.01	0.98	0.97	0.96	0.93	0.99	1.03	0.97	1	0.99	0.99
	Reference - 319nt	0.99	1.01	1.02	1.01	0.99	1	0.98	1.02	0.95	1.03	1.02	1.03	1.03	1.01	0.97	1.05	1	0.99	1.01
	Reference - 283nt	1	1	1.02	1	1	0.99	1	1.02	1.02	1	1	1	0.98	1	0.98	1	1.01	1.02	0.98
**Reference probes**	Reference - 256nt	1	1	0.95	0.96	1.05	1.01	0.99	0.95	0.99	0.94	0.99	1	0.98	0.95	1.02	0.97	0.93	0.96	1
