# Supplementary material for: Whole genome sequencing identifies novel structural variant in a large Indian family affected with X-linked agammaglobulinemia
Source: PLoS One. 2021 Jul 12;16(7):e0254407. doi: 10.1371/journal.pone.0254407 (PMC8274882; doi:10.1371/journal.pone.0254407)
Supplement: S1 File — (DOCX) [file pone.0254407.s007.docx]

**S1 File**

**Supplementary Data 1**

**Detailed clinical features of XLA large deletion family.**

**Patient 1 (P1-V14)**

The first patient was born to non-consanguineous parents after the father’s second marriage following the death of his first wife. At the time of admission to the clinic, he was 8 years old with recurrent respiratory infections since infancy, recurrent pyoderma for two years and grade 1 clubbing with empty tonsillar fossae. He had a positive primary immunodeficiency family history, with two male sibling deaths. Immunoglobulin assay showed an IgA level of 0.2 g/l, IgG of 3 g/l. and IgM of 0.31 g/l. Flow cytometry assay revealed null CD19^+^/CD20^+^ B lymphocyte count as 0% i.e. 0/mm^3^ where biological reference was 270-860/mm^3^. It also revealed increase in CD3^+^, CD3^+^/CD4^+^, and CD3^+^/CD8^+^ T lymphocyte count as 91% i.e. 6458/mm^3^, 37% i.e. 2626/mm^3^ , and 47% i.e. 3335/mm^3^ where biological reference was 570-2400, 430-1800 and 210- respectively. It showed normal CD3+/CD16+56+ T lymphocytes as 5% i.e. 355/mm^3^ where normal biological reference was 78-470. Investigations are tabulated in Table 1. He was suspected to have XLA. However, the father refused treatment with IVIG and he is being treated by practitioners of alternative systems of medicine.

**Patient 2 (P2-V18)**

A second patient was the first male twin child of healthy non-consanguineous parents. At the time of admission, the child was 2.5 years old, with weight faltering, suffering from recurrent oral thrush, recurrent diarrhea and acute suppurative otitis media with onset from 5 months of age.. There was a positive primary immunodeficiency family history, where the first cousin had expired due to XLA and his elder sister's children had a history of recurrent infections. Flow cytometry assay revealed null CD22^+^ mature B cells as 0% i.e. 0/mm^3^ and increase in CD3^+^ T cell lymphocyte of 94% i.e. 5475/mm^3^ where biological reference was 390-1400/mm^3^ and 1460-5440/mm^3^ respectively. Immunoglobulin assay revealed panhypogammaglobulinemia . Investigations are tabulated in Table 1. He was also suspected to have XLA and was started on prophylaxis with monthly IVIG and cotrimoxazole.

**Patient 3 (P3-V22)**

This was a one and a half - year - old male baby, born at term with a normal birth weight to non - consanguineous parents who presented with bronchopneumonia at the same time as his first cousin, patient 2..He had a history of recurrent pneumonia, diarrhea and pyoderma from seven months of age.He was wasted with weight for height less than the third centile and had absent tonsils and non - palpable cervical lymph nodes.He had hypogammaglobulinemia with IgG level of 1.20 g/l (Reference range - 4.07-10.09 g/l ) and negligible B cells on lymphocyte subset analysis of 8 cells/cu.mm (Normal range - 430-3300/ cu.mm). He was started on prophylaxis with IVIG with a provisional diagnosis of X - linked agammaglobulinemia considering the positive family history of other male children with a similar history, but developed seizures with fever two months later and succumbed to the illness which was diagnosed as meningitis.

**Patient 4 (P4-V23)**

The fourth patient was born of non-consanguineous parentage. At the time of admission to the clinic, he had a history of recurrent diarrhea for the past one month.. He was 6 months old and had a positive primary immunodeficiency family history, having lost an elder sibling with probable X linked agammaglobulinemia.( Patient 3 ). He had negligible CD19^+^ B cell lymphocyte count as 0.2% i.e. 10/mm^3^ revealed by flow cytometry report, its biological reference was 610-2600/mm^3^. Flow cytometry assay also revealed normal count of CD3^+^, CD3^+^/CD4^+^, CD3^+^/CD8^+^, and CD3^+^/CD16^+^56^+^ T lymphocyte count as 82% i.e. 4169/mm^3^, 62% i.e. 3152/mm^3^, 19% i.e. 966/mm^3^, and 13% i.e. 661/mm^3^ where biological reference for these cells were 2170-6500, 1580-4850, 680-2470 and 80-340 respectively. Investigation reports are tabulated in Table 1. A provisional diagnosis of XLA was made.The child underwent a hematopoietic stem cell transplant (HSCT) from a matched sibling donor.

**Patient 5 (P5-IV101)**

This seventeen year old boy, the second son born to non - consanguineous parents had a history of recurrent pneumonia, diarrhea and pyoderma and one episode of pyogenic meningitis with onset of symptoms at six months of age.Three of his maternal uncles had died in childhood.There was a history of four male children in the family being diagnosed to have X - linked agammaglobulinemia represented in Figure 1.He was found to have low immunoglobulin levels and evidence of bronchiectasis on HRCT at seven years of age and advised monthly immunoglobulin prophylaxis.However, the family did not comply with the advice and he was given prophylactic antibiotics alone. He was lost to follow up and presented ten years later with features of bronchiectasis and arthritis of both knees. Flow cytometric analysis revealed total absence of B cells and a diagnosis of X - linked agammaglobulinemia was confirmed.
